# Urinary continence outcomes, surgical margin status, and complications after radical prostatectomy in 2,141 German patients treated in one high-volume inpatient rehabilitation clinic in 2022

**DOI:** 10.1007/s00345-024-05200-0

**Published:** 2024-08-22

**Authors:** Henning Bahlburg, Patricia Rausch, Karl Heinrich Tully, Sebastian Berg, Joachim Noldus, Marius Cristian Butea-Bocu, Burkhard Beyer, Guido Müller

**Affiliations:** 1 Department of Urology, Marien Hospital Herne, Ruhr-University Bochum, Hölkeskampring 40, 44625 Herne, Germany; 2 Center for Urological Rehabilitation, Kliniken Hartenstein, Bad Wildungen, Germany

**Keywords:** Radical prostatectomy, Urinary incontinence, Nerve-sparing surgery, Diabetes mellitus, Age

## Abstract

**Purpose:**

To identify independent predictors of urinary continence and report early complications after radical prostatectomy (RP) in a large, contemporary German cohort.

**Methods:**

Urinary incontinence data of patients undergoing 3-week inpatient rehabilitation (IR) after RP were prospectively assessed by 24-hr pad test and uroflowmetry at the beginning and the end of IR, respectively. Lymphoceles were assessed prospectively by ultrasound. Tumor and patient characteristics, and information on urinary leakage on initial cystography were retrospectively extracted from discharge letters and surgical reports. Regression analyses were performed to identify predictors of urinary continence at the beginning of IR.

**Results:**

Overall, 2,141 patients were included in the final analyses. Anastomotic leakage on the initial cystography and lymphoceles were found in 11.4% and 30.8% of patients, respectively. Intervention for a symptomatic lymphocele was required in 4.2% of patients. At the end of IR, 54.2% of patients were continent, while the median urine loss decreased to 73 g (interquartile range 15–321). Multivariable logistic regression analysis identified age and diabetes mellitus as independent negative predictors, but nerve-sparing surgery as an independent positive predictor of urinary continence (each *p* < 0.001). Multivariable linear regression analysis showed that 24-hr urine loss increased by 7 g with each year of life (*p* < 0.001), was 79 g higher in patients with diabetes mellitus (*p* = 0.007), and 175 g lower in patients with NS (*p* < 0.001).

**Conclusion:**

Age, diabetes mellitus, and NS are significantly associated with continence outcomes in the early period after RP. Our analyses may help clinicians to pre-operatively counsel patients on potential surgical outcomes.

## Introduction

Urinary incontinence after radical prostatectomy (RP) for prostate cancer (PCa) can severely influence the quality of life [1–4], may contribute to decision regret, and even lead to depression [5]. The “pentafecta” (negative surgical margins, urinary continence, potency, cancer control, no complications) are established as the most important quality control measures of RP [6]. The importance of nerve-sparing surgery (NS) on functional outcomes is described extensively in the literature [7–9]. Higher surgical experience and treatment in certified cancer centers have previously been associated with improved functional and oncological outcomes [10–12]. As highlighted by a recent reverse systematic review by Moretti et al. including 193,618 patients after either open RP (ORP), laparoscopic RP or robot-assisted RP (RARP), urinary continence are comparable between the surgical approaches [13]. Urinary continence outcomes are also known to be associated with the patients’ age as recently described in two multicenter studies conducted by Gondoputro et al. [14] and Holze et al. [15], respectively. Additionally, urinary continence in the early period after RP may also negatively be influenced by the presence of diabetes mellitus, as described by Nishikawa et al. [16] and Cakmak et al. [17] reporting on > 300 patients each. Lymphoceles are common after RP and may cause further complications (deep venous thromboembolism, infections) in 2–15% of cases [18–20]. Leakage of the urethro-vesical anastomosis is found in 0.3–15.4% of patients with no differences between ORP and RARP [21, 22]. Positive surgical margins on final histopathology are influenced by the surgeons’ [23] and the pathologists’ [24] experience, as well as tumor characteristics (Gleason score, tumor stage, PSA-levels) [24, 25]. Ultimately, cancer-specific mortality, overall mortality, and biochemical recurrence free survival are significantly influenced by positive surgical margins [25, 26].

Supporting patients to reach the critical goal of reintegration into daily life, German social laws entitle PCa patients to an average of three weeks of inpatient rehabilitation (IR) after RP. Furthermore, the guideline of the German Society of Urology recommends that all patients be offered several weeks of IR after RP to minimize functional and psychosocial disorders [27]. The costs for this expensive endeavor are covered by social insurances (i.e. retirement funds).

In this study, we aim to identify independent predictors of urinary continence in the early postoperative period in a contemporary cohort of patients referred to IR after RP. Furthermore, we aim to analyze potential differences in positive surgical margins, tumor characteristics, surgical approach (ORP vs. RARP), and early complications (e.g., lymphoceles, anastomotic leakage) between centers. The IR setting including patients from all over Germany and all levels of care allows for an impartial third-party evaluation of outcomes in the early period after RP.

## Methods

### Data collection

This study is based on data collected from all patients with PCa after RP undergoing IR in a specialized center for urological rehabilitation (Kliniken Hartenstein, Bad Wildungen, Germany) between January and December 2022.

All patients gave their informed consent prior to data collection. The study was approved by the appropriate ethics board (2024-3675-evBO). Information on tumor characteristics, surgical approach, NS, and anastomotic leakage on initial cystography were retrospectively extracted from discharge letters and, if available, surgical reports. The presence of lymphoceles was prospectively assessed by ultrasound at the beginning of IR. If lymphoceles were detected, ultrasound was repeated once weekly or when patients reported symptoms such as fever, pain in the pelvic area, or leg swelling. If necessary, computer tomography- or ultrasound-guided drainage of symptomatic lymphoceles was performed. The final analyses focused on patients from primary hospitals with a referral rate of ≥ 50 patients/year. Incontinence and micturition volume were prospectively assessed by 24-hr pad test and uroflowmetry at the beginning (T1) and end (T2) of IR. Continence was defined as no pad use or no urine loss at the 24-hr pad test. Baseline characteristics included patient age, PSA levels before surgery, tumor stage, surgical approach (ORP vs. RARP), and utilization of NS.

### Inpatient rehabilitation

During IR, patients were routinely seen by physicians and were treated daily by specialized physiotherapists regarding urinary continence. The multimodal continence therapy developed at Kliniken Hartenstein includes osteopathic physiotherapy and external urethral sphincter exercises. For patients without improvement in daytime continence within two weeks of therapy, video-assisted biofeedback sphincter training via transurethral endoscopy may be performed. Patients suffering from severe urge incontinence were given anticholinergic drugs to reduce postoperative de novo detrusor instability [28].

### Statistical analysis

Descriptive statistics for categorical variables included frequencies and proportions, while for continuous variables medians and interquartile ranges (IQR) were reported. Between-group comparisons were analyzed using the Mann-Whitney U test or a Chi-squared test (Pearson) as appropriate. The Wilcoxon test was used to compare changes in quantitative variables, while a Chi-squared test (McNemar) was used to compare changes in proportions. Uni- and multivariable regression analyses were performed to identify independent predictors of urinary continence and factors influencing urine loss in the 24-hr pad test in incontinent patients, respectively.

## Results

Overall, 3,751 patients from 213 primary hospitals underwent IR after RP in one institution in 2022. Of these, 2,141 patients were referred by 21 different primary hospitals with an annual referral volume of ≥ 50 patients. IR started at a median of 21 days (inter-quartile range (IQR) 18–27) after RP and ended at a median of 42 days (IQR 38–49) after RP. The results revealed variability in the surgical approach (ORP vs. RARP), postoperative complications, and oncological outcomes in different PCa centers as demonstrated by the minimum and maximum values presented (Table 1). Overall, RARP and NS were performed in 89.3% and 73.7% of patients, respectively. Locally advanced tumor stage (≥ pT3), Gleason sum score ≥ 8, and lymph node metastases were present in 36.5%, 18.2%, and 9.6% of patients, respectively. Positive surgical margins on final histopathology were found in 5.5% and 29.8% of patients with tumor stages ≤ pT2 and ≥ pT3, respectively. Anastomotic leakage on the initial cystography with subsequent longer catheter indwelling times and lymphoceles were found in 11.4% and 30.8% of patients, respectively. An intervention for a symptomatic lymphocele was necessary in 4.2% of patients (*n* = 89).

**Table 1:** Baseline characteristics of 2,141 patients after radical prostatectomy with the corresponding range of 21 different primary hospitals

Variable	Total	Range	
		Minimum	Maximum
Age (years), Median (IQR)	67 (62–72)	63 (60–69)	69.5 (65–73)
PSA (ng/ml), Median (IQR)^a^	7.60 (5.5–11.3)	6.6 (4.7–9.3)	9.7 (6.9–15.3)
BMI (kg/m^2^), Median (IQR)	26.8 (24.5–29.4)	26.0 (23.6–27.6)	27.8 (23.4–31.0)
≥ 30 (%)	25.4	17.5	36.1
Cardiovascular disease (%)	63.2	47.1	81.0
Diabetes (%)	10.8	2.6	22.4
Robot-assisted approach (%)	89.3	0	100
Nerve-sparing, *n* (%)^b^	73.7	21.2	95.2
Tumor stage ≥ pT3 (%)^c^	36.5	19.7	52.4
Lymph node positive (%)^d^	9.6	1.6	21.1
Positive surgical margin (R1)^e^	14.3	3.5	33.8
R1 ≤ pT2 (%)	5.5	0.0	21.6
R1 ≥ pT3 (%)	29.8	8.2	63.2
GS ≥ 8 (%)^f^	18.2	10.4	48.1
Lymphocele (%)	30.8	13.8	52.0
Leak of urinary anastomosis (%)	11.4	1.7	26.6

**Abbreviations**:

IQR = interquartile range

BMI = body mass index

^a^ data available for *n* = 2015

^b^ data available for *n* = 2123

^c^ data available for *n* = 2087

^d^ data available for *n* = 2076

^e^ data available for *n* = 2065

^f^ data available for *n* = 2043

The “minimum” column represents the smallest value (median or percentage) reached by a single institution, the “maximum” column depicts the highest value (median or percentage) identified for that respective characteristic. For example, patients from one center reported a median age of 63 years (interquartile range 60–69; minimum), while patients from a different center reported a median age of 69.5 years (interquartile range 65–73; maximum)

### Urinary continence outcomes in the early period after RP

**Fig. 1:**
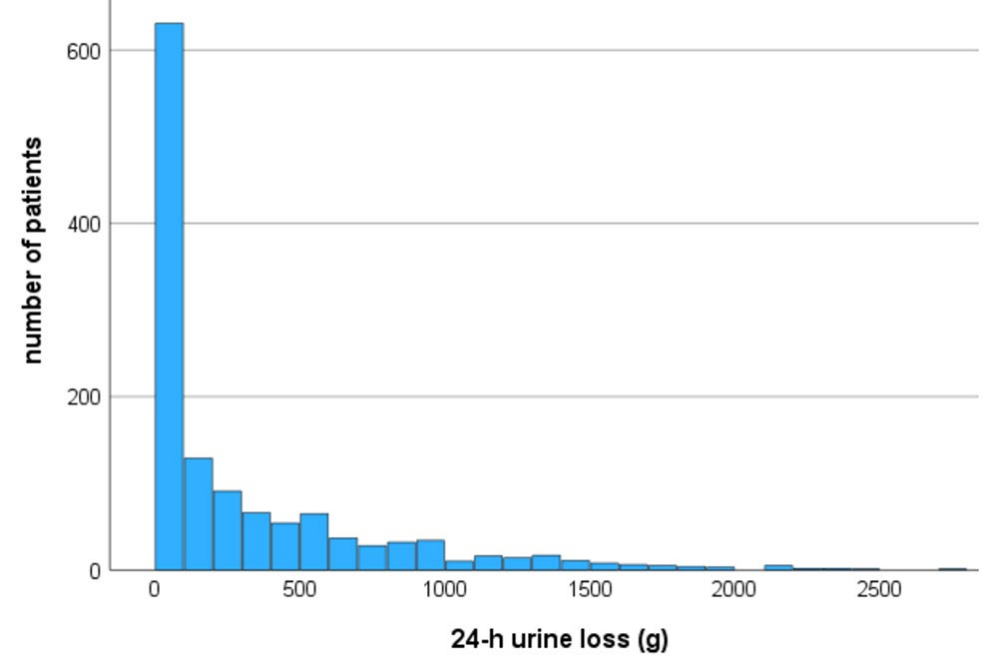
Distribution curve for urine loss in the 24-hr pad test at the beginning of inpatient rehabilitation (median 21 days (IQR 18–27) after radical prostatectomy)

Urinary continence at T1 was reported by 36.6% of patients. The graph shows a left-skewed distribution of urine loss in the 24-hr pad test (Fig. 1) with a median loss of urine at T1 of 102 g/day (IQR 15–463). At T2, the rate of continent patients improved to 54.2% (*p* < 0.001) and 24-hr urine loss decreased to 73 g/day (IQR 15–231; *p* < 0.001), respectively. The median micturition volume increased significantly during IR (T1: 170 ml (IQR 103–256) vs. T2: 218 ml (IQR 146–298), *p* < 0.001).

### Regression analyses to identify predictors of urinary continence at the beginning of IR

Univariable logistic regression analysis identified younger age (odds ratio (OR) 0.938, 95% confidence interval (CI) 0.925–0.951, *p* < 0.001), cardiovascular disease (OR 0.739, 95% CI 0.616–0.887, *p* < 0.001), diabetes mellitus (OR 0.486, 95% CI 0.352–0.672, *p* < 0.001), RARP (OR 1.745, 95% CI 1.276–2.387, *p* < 0.001), and NS (OR 3.210, 95% CI 2.537–4.061, *p* < 0.001) as predictors of urinary continence at the beginning of IR (Table 2a). On multivariable logistic regression analysis, only age (OR 0.950, 95% CI 0.937–0.964, *p* < 0.001) and diabetes mellitus (OR 0.564, 95% CI 0.401–0.793, *p* < 0.001) were identified as independent negative predictors, while NS (OR 2.824, 95% CI 2.201–3.625, *p* < 0.001) was identified as the only independent positive predictor for urinary continence at the beginning of IR, respectively (Table 2b).

**Table 2:** Uni- (**a**) and multivariable (**b**) logistic regression analysis to identify predictors of urinary continence at the beginning of inpatient rehabilitation (3 weeks after surgery)

Variable	(a) univariable		(b) multivariable	
	OR (95% CI)	*p*	OR (95% CI)	*p*
Age (continuous)	0.938 (0.925–0.951)	**< 0.001**	0.950 (0.937–0.964)	**< 0.001**
CVD	0.739 (0.616–0.887)	**< 0.001**	0.895 (0.733–1.092)	0.273
Diabetes	0.486 (0.352–0.672)	**< 0.001**	0.564 (0.401–0.793)	**< 0.001**
BMI ≥ 30 kg/m^2^	1.000 (0.814–1.228)	0.998	1.063 (0.853–1.325)	0.584
Robot-assisted approach	1.745 (1.276–2.387)	**< 0.001**	1.269 (0.908–1.773)	0.163
Nerve-sparing procedure	3.210 (2.537–4.061)	**< 0.001**	2.824 (2.201–3.625)	**< 0.001**

**Abbreviations:**

CVD = cardiovascular disease

BMI = body mass index

OR = odds ratio

**Bold** font indicates significant results

### Regression analyses to identify predictors of urinary loss at the beginning of IR

Univariable linear regression analysis identified age (regression coefficient (r) = 9.8, 95% CI 7.3–12.4, *p* < 0.001), cardiovascular disease (*r* = 40.6, 95% CI 2.9–78.2, *p* = 0.035), diabetes mellitus (*r* = 112, 95% CI 54.2–170.6, *p* < 0.001), RARP (*r*=-63.6, 95% CI -121.9–-5.2, *p* = 0.033), and NS (*r*=-198.3, 95% CI -237.7–-158.9, *p* < 0.001) to significantly influence urine loss in the 24-hr pad test in incontinent patients at T1 (Table 3a). At this point, increasing age (*r* = 7.0, 95% CI 4.3–9.6, *p* < 0.001) and presence of diabetes mellitus (*r* = 78.6, 95% CI 21.4–135.9, *p* = 0.007) contribute negatively to urine loss, while NS (*r*=-175.3, 95% CI -216.2–-134.4, *p* < 0.001) was the only variable identified to positively impact urine loss in the 24-hr pad test in incontinent patients at the beginning of IR by multivariable linear regression analysis (Table 3b). Accordingly, 24-hr urine loss increased by 7 g with each year of life, was 79 g higher in patients with diabetes mellitus, and 175 g lower in patients with NS.

**Table 3:** Uni- (**a**) and multivariable (**b**) linear regression analysis to identify predictors and their impact on urine loss during the 24-hour pad-test at the beginning of inpatient rehabilitation (3 weeks after surgery)

(a) univariable	***t***	regression coefficient	95% CI	*p*
Age (continuous)	7.5	9.8	7.3 to 12.4	**< 0.001**
CVD	2.1	40.6	2.9 to 78.2	**0.035**
Diabetes mellitus	3.8	112.4	54.2 to 170.6	**< 0.001**
BMI ≥ 30 kg/m^2^	-0.4	-8.3	-50.1 to 33.5	0.696
Robot-assisted approach	-2.1	-63.6	-121.9 to -5.2	**0.033**
Nerve-sparing surgery	-9.9	-198.3	-237.7 to -158.9	**< 0.001**
**(b) multivariable**	***t***	**regression coefficient**	**95% CI**	***p***
Age (continuous)	5.2	7.0	4.3 to 9.6	**< 0.001**
CVD	0.7	12.6	-25.0 to 50.2	0.512
Diabetes mellitus	2.7	78.6	21.4 to 135.9	**0.007**
BMI ≥ 30 kg/m^2^	-0.7	-14.6	-55.4 to 26.2	0.483
Robot-assisted approach	-0.3	-8.5	-66.1 to 49.2	0.774
Nerve-sparing surgery	-8.4	-175.3	-216.2 to -134.4	**< 0.001**

**Abbreviations**:

CI = confidence interval

CVD = cardiovascular disease

BMI = body mass index

**Bold** font indicates significant results

## Discussion

In patients suffering from urinary incontinence in the early period after RP, increasing age (*r* = 7.0, 95% CI 4.3–9.6, *p* < 0.001) and presence of diabetes mellitus (*r* = 78.6, 95% CI 21.4–135.9, *p* = 0.007) contribute negatively to urine loss, while NS (*r*=-175.3, 95% CI -216.2–-134.4, *p* < 0.001) was the only variable identified to positively impact urine loss in the 24-hr pad at T1.

A previous analysis of 2,998 patients (∼ 90% ORP, ∼ 10% RARP) from one German highest-volume institution spanning the years 2003–2013 also identified age and NS as independent predictors of urinary continence at the end of 3 weeks of IR, while diabetes mellitus was not significantly associated with urinary continence at this point [7]. In this single-center study spanning 10 years, RARP was associated with significantly improved urinary continence in the early period after RP. Meanwhile, in our cohort including patients from 21 primary hospitals undergoing RP in one year, surgical approach was only identified to contribute to urinary loss in incontinent patients in univariable, but not in multivariable linear regression analysis. Diabetes mellitus has previously been identified to significantly influence continence rates after RARP [16, 17], albeit in much smaller cohorts than presented in this study. As polyneuropathy is the most prevalent complication of diabetes mellitus [29], a negative impact on small nerve fibers innervating the urethral sphincter with subsequent impaired function seems plausible.

The influence of NS on functional outcomes after RP has been highlighted several times [6, 8]. As information on NS was taken from discharge letters and, if available, surgical reports, a distinction between uni- or bilateral nerve-sparing was not feasible in all patients. Nonetheless, our results corroborate previous findings and again emphasize the importance of NS on functional outcomes after RP. The impact of age on early urinary continence has also previously been highlighted [14, 15]. In our analysis, BMI ≥ 30 kg/m^2^ was not associated with early continence outcomes. A correlation between obesity and long-term urinary continence outcomes is well-described [30–32]. However, in line with our results, no such correlation was found between obesity and short-term urinary continence outcomes after RP [33, 34].

Positive surgical margins on final histopathology in both tumor stages ≤ pT2 and ≥ pT3 were found in 5.5% and 29.8% of patients, respectively. It is known that intraoperative whole-mount sections improve NS and oncological outcomes, since remaining PCa may be detected and resected subsequently [8]. However, in more rural areas intraoperative whole-mount sections face logistical challenges and may thus not be the standard of care. Lymphoceles were found in 30.8% of all patients, which is in line with the literature [35–37]. In our cohort, 4.2% of patients (*n* = 89) required an intervention for a symptomatic lymphocele, which is within the range of 0–8% of symptomatic lymphoceles described in a systematic review by Ploussard et al. [38]. Furthermore, the presence of anastomotic leakage on initial cystography in 11.4% of patients is in line with previously reported data [21, 22].

Since there are several dedicated urological rehabilitation centers in Germany, primary hospitals may refer their patients to more than one center. Additionally, the annual surgical volume per center is unknown to us. Accordingly, no final conclusions can be drawn about the relationship between surgical volume per center and functional and/or oncological outcomes and complications. The Center for Urological Rehabilitation at Kliniken Hartenstein (Germany’s largest urological IR center per annual patient volume) offers unique expertise and experience and a selection bias in patients referred to this institution cannot be ruled out. IR is unique to the German healthcare system and therefore enables the analysis of surgical outcomes in a large number of patients from all levels of care within a short time frame. However, as IR is cost-intensive and well-established, comparable studies in different healthcare systems are virtually impossible.

Despite its limitations, our study provides real-world data on continence outcomes, complications, and surgical margin status in > 2,000 patients from all over Germany treated in one specialized urological IR center in 2022. By including patients from all levels of care, we expect these results to be representative of current nationwide treatment patterns and surgical outcomes after RP. Our data not only emphasize the importance of NS but also delineate the role of diabetes mellitus and age in early urinary continence after RP. These results may help clinicians identify patients at risk for prolonged urinary incontinence and potentially guide affected patients to intensified urethral sphincter exercises to restore continence.

## Conclusion

Age, diabetes mellitus, and NS are significantly associated with both urinary continence and urine loss in the early period after RP. Our analyses may help clinicians pre-operatively counsel patients on potentially decreased urinary continence. The data presented offer the opportunity to evaluate each center’s outcomes within a comparative framework.

## Data Availability

Data are not publicly available but may be made available by the corresponding author upon reasonable request.
